# Human Factors Considerations and Metrics in Shared Space Human-Robot Collaboration: A Systematic Review

**DOI:** 10.3389/frobt.2022.799522

**Published:** 2022-02-03

**Authors:** Sarah Hopko, Jingkun Wang, Ranjana Mehta

**Affiliations:** Neuroergonomics Laboratory, Department of Industrial and Systems Engineering, Texas A&M University, College Station, TX, United States

**Keywords:** trust, workload, situation awareness, heart rate variability, survey, physical human-robot interaction

## Abstract

The degree of successful human-robot collaboration is dependent on the joint consideration of robot factors (RF) and human factors (HF). Depending on the state of the operator, a change in a robot factor, such as the behavior or level of autonomy, can be perceived differently and affect how the operator chooses to interact with and utilize the robot. This interaction can affect system performance and safety in dynamic ways. The theory of human factors in human-automation interaction has long been studied; however, the formal investigation of these HFs in shared space human-robot collaboration (HRC) and the potential interactive effects between covariate HFs (HF-HF) and HF-RF in shared space collaborative robotics requires additional investigation. Furthermore, methodological applications to measure or manipulate these factors can provide insights into contextual effects and potential for improved measurement techniques. As such, a systematic literature review was performed to evaluate the most frequently addressed operator HF states in shared space HRC, the methods used to quantify these states, and the implications of the states on HRC. The three most frequently measured states are: trust, cognitive workload, and anxiety, with subjective questionnaires universally the most common method to quantify operator states, excluding fatigue where electromyography is more common. Furthermore, the majority of included studies evaluate the effect of manipulating RFs on HFs, but few explain the effect of the HFs on system attributes or performance. For those that provided this information, HFs have been shown to impact system efficiency and response time, collaborative performance and quality of work, and operator utilization strategy.

## 1 Introduction

The improvement of robot automation in the manufacturing process has made it increasingly possible to incorporate the advantages of operators in shared space human-robot collaboration (HRC). There are benefits of automated production systems as robots can manipulate heavy payloads, perform repetitious tasks, and work in unsafe environments in place of the human, and the utilization of robots can increase system productivity and product quality for lower unit prices (Villani et al., 2018), (Vysocky and Novak, 2016). However, complete automation is not always feasible due to the uncertainty of workpiece conditions, handling limits of the robots, difficulties with sensing products, or needed customizability based on consumer demands (Pagilla and Yu, 2001; Mital and Pennathur, 2004; Bogue, 2009; Niknam et al., 2018). In conditions such as these, operators provide the advantage of increased recognition, flexibility, and creative decision-making under uncertain environments. Thus, to optimize the production process and associated advantages of humans and robots, the idea of human-robot collaboration has emerged. Within shared space HRC, collaborative robots (cobots) are intelligent assist robots designed for direct, physical interaction (Akella et al., 1999). The level of collaboration between a cobot and operator tends to increase as the proximity between the entities reduces (Vysocky and Novak, 2016); however, shared environments and physical interaction can often lead to decreased system safety, decreased performance, and decreased efficiency resulting from a lack of a systems perspective that accounts for emergent human factors such trust, anxiety, increased mental strain or workload, and the corresponding utilization of the technology (Lee and Seppelt, 2009; Fujita et al., 2010; Charalambous et al., 2016).

While the shared space nature of HRC allows for improved collaboration, the safety-critical nature of shared space work is an important concern that influences operator experience: in the manufacturing sector, accidents have occurred between operators and robots, where collision between the agents have resulted in large-scale damage to equipment, disruption of manufacturing processes, and even worker fatalities (Bryant, 2015), (Naderpour et al., 2015). Collision avoidance literature focuses on adaptive robotics and equipping cobots with new sensors and emergency stops; however, the human component itself can induce decreased safety due to poor situation awareness, low maintenance on the cobot increasing the likelihood of system failure, and overreliance and abuse of the cobot to perform perfectly (Mital and Pennathur, 2004), (Lee and See, 2004). Furthermore, human factor considerations are required for improving performance in advanced manufacturing. The introduction of humans into the manufacturing workcell alongside robots has implications on the emergent properties associated with human-robot interaction and the implications of operators’ states. A common view is that increased collaboration with robots and automation can prevent the performance decrements associated with human error by eliminating the variability in human performance and reducing the workload placed on the operator (Lee and Seppelt, 2009). However, collaboration in HRC can often induce higher workload and decrease human performance if the implementation of HRC does not consider the resulting impact of automation on the operator. The design of collaborative systems needs to consider the implications of automation on both the robot’s performance and the human’s performance to optimize the overall system benefits.

Many automated manufacturing systems place the human in a passive, supervisory role while the automation performs under certain, uniform environments. As such, the human is then responsible for monitoring for off nominal instances and compensating for the unexpected, and, in shared-space collaboration, is at risk of losing situation awareness of the cobot, increasing the chance of collision. This type of role can result in poor human performance as supervisory roles pull the human out-of-the-loop, decreasing their situation awareness and engagement in the task, and preventing them from easily resuming control (Kaber and Endsley, 2004). It is easy to blame the human operator for “human error” when they miss detect an off-nominal instance; however, poor design of the automated system and associated interactions result in less-than-optimal compensatory responses from the human. Additionally, as automation is often designed to take over predictable parts of the tasks, the workload placed on the operator can increase as the remaining instances can be more difficult, demanding, and unpredictable on the operator to resolve. Thus, collaboration with automation and robots and associated functional allocations of roles and responsibilities should be jointly determined on both the human factors and robot factors, and their key interactions.

### 1.1 Study Objectives

While the study of human factors (HFs) in automation is long reaching (Lee and Seppelt, 2009), (Madhavan and Wiegmann, 2007), (Hancock et al., 2011), few studies systematically investigate these factors for a shared-space work environment, where perceived safety of the operator is highly relevant and new interaction modalities are present, or for cases of active collaborative teaming with robots. For those that consider HFs, HFs are primarily regarded as dependent variables influenced by the collaborative system or by robot factors. Thus, the implications of HFs themselves and their potential interaction with other HFs on metrics of HRC has not be systematically documented for such environments. Moreover, given the increased motivation for human factor works within shared space HRCs, the state-of-the-art methods to capture the HFs similarly need to be documented as these methodological measurements can provide contextual insights into the HFs and can provide insight into improved measurement techniques. As such, the primary goals of this study are to: 1) systematically document examined human factors within shared space HRC and the associated methods employed to quantify these factors, 2) summarize the implications of the identified HFs on metrics of HRC and 3) develop recommendations for robotic cognitive support given these factors to improve the metrics of HRC.

## 2 Methods

A systematic review was performed to address the goals of the study. Inclusion criteria search terms were broken into two groups: operator state and shared space HRC (Table 1) in addition to all related words and equivalent subjects. Key terms were identified through background reviews, where different spellings, tenses, and variants were also included. These terms were searched for in either the title, abstract, or keywords of a paper using Boolean AND logic between groups and Boolean OR logic within each group; one element of each group must have appeared for it to have been considered. Additional inclusion criteria consisted of papers written in English, scholarly (peer-reviewed) papers including conference proceedings but excluding preprints, and papers published between 2000–2020. The search was conducted on April 8th, 2020, using database specific searches and EBSCO, an engine with access to over 200 + databases in addition to Texas A&M University’s library and associated subscriptions. EBSCO is an adaptive search that applies the inclusion keywords twice: once to filter through relevant databases and journals and again to the actual article. EBSCO filters twice, so it has a higher chance of missing papers that are included in journals that do not traditionally overlap with the search’s keywords, although it can pull from more obscure journals that are in less used databases. To mitigate the potential for missing papers, additional databases were also searched directly: PubMed, MEDLINE, Engineering Source, Applied Science and Technology Source, Academic Search Ultimate, and Compendex (Engineering Village).

**TABLE 1:** Search terms.

Group	Search terms
HRC	human-robot co-manipulation, human-robot collaboration, human-robot cooperation, co-bots, cobots, cooperative robots, collaborative robots, human-robot interaction
Operator State	acceptance, fatigue, stress, frustration, trust, safety, mental, exhaustion, anxiety, arousal, cognition, workload, sleep, psychological, worker state, awareness

In addition to search terms, all variations, synonyms, and equivalent subjects were included.

**FIGURE 1:**
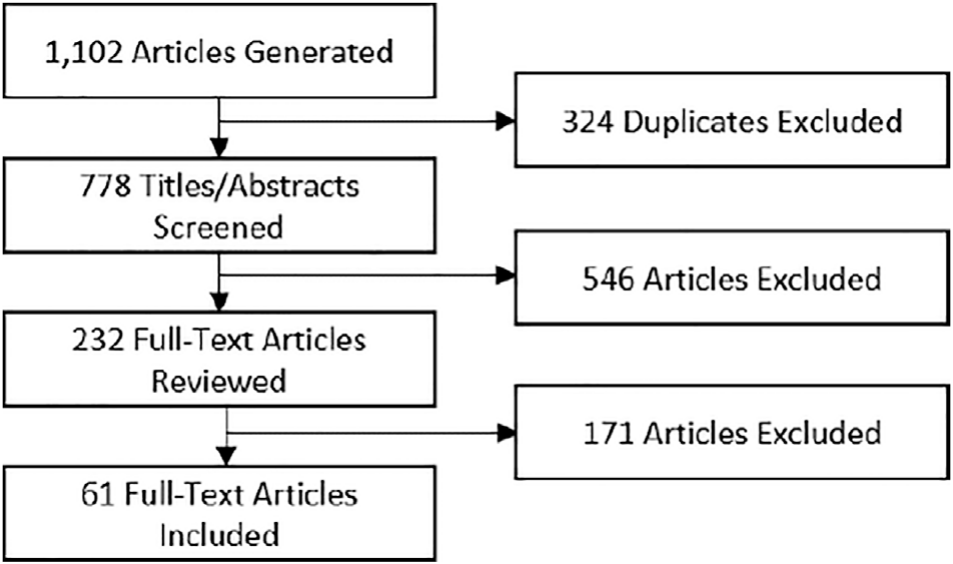
Systematic review flow diagram.

The accumulative search resulted in 1,102 articles. A standard systematic review method was used to review the articles for fit (Figure 1). All title and abstracts were blind reviewed by at least two researchers. All (included/excluded) conflicts were discussed between the reviewers before proceeding to full paper reviews. The following exclusion criteria were applied. First, the article must have been related to shared space human-robot collaboration. Collaboration is here defined as robotics that are designed for direct teaming in a shared workspace and can operate without a host: exoskeletons, wearables, and non-shared space teleoperated robots are thus excluded. Exoskeletons and wearables are not here considered teaming robotics as they are currently utilized as tools rather than teammates and cannot be used in any context without the human host. This type of interaction has differently relevant factors of importance and different types of interaction modes. Teleoperated robotics are included so long as they are operated within the same workspace as the human. Second, the article must have addressed a human physiological or psychological factor with respect to the collaborative task. Papers that focused exclusively on human movement dynamics or position for collision avoidance, or for other similar safety purposes were excluded. Third, the article must have been an application of an experimental design that includes measurement of the human factor; as such literature reviews and theory/formulation papers were excluded. No identified literature reviews explicitly reviewed experimental methods. Sixty-one papers were included in the final synthesis meeting these criteria.

## 3 Results

The following sections outline the extracted results from the included studies including: 1) participant demographic norms (i.e., sample size, average age, gender), 2) experimental training norms, 3) the human factors addressed in the studies (either through manipulation or measurement), 4) the specific measurement and manipulation methods for each human factor, and lastly 5) the resulting implications of the human factors on metrics of HRC.

### 3.1 Participant Demographic Norms

Forty-nine of the sixty-one papers included information about the number of participants (Table 2). On average, each study recruited 20.76 (16.05) participants after excluding four statistical outliers with 90, 115, 208, and 231. These four outliers were survey or convention populations (Maurtua et al., 2017; Chen et al., 2018, 2020; Eimontaite et al., 2019). Thirty-five papers reported gender distribution; excluding the outliers, the average number of male, female participants were 11.94 (6.82) and 7.03 (4.95), respectively. The average age of participants was found to be 27.41 (4.64), which is calculated by a weighted average age for each study sample. Additionally, only thirteen of the sixty-one papers explicitly reported participants’ prior experience with collaborative robotics; however, an additional nine papers mentioned that participants were engineering students or faculty.

**TABLE 2:** Participant demographic summary.

Variable	*n*	Mean	SD	Min	Max
Sample Size	49	20.76	16.05	1	63
Avg. Age	29	27.41	4.64	20	42.30
# Male	35	11.94	6.82	1	26
# Female	29	7.03	4.95	1	18
Gender Ratio	38	0.51	0.41	0	1.86

This table illustrates the distributions of participant demographics across the included papers. Not every study provided distribution information, hence “*n*” reported. The gender ratio is calculated by dividing #Female by #Male individual for every study.

### 3.2 Participant Training Norms

Only eighteen of the sixty-one papers mentioned their training processes for the participants. Four papers reported providing unlimited training—where participants were introduced to the experiments and allowed to practice until they were satisfied. Eleven papers had limited training—they either used examples, which simplified the experiments, or limited total practicing time. The remaining three papers provided overviews or videos, but no hand-on experiences to the participants.

### 3.3 Addressed Human Factors

**FIGURE 2:**
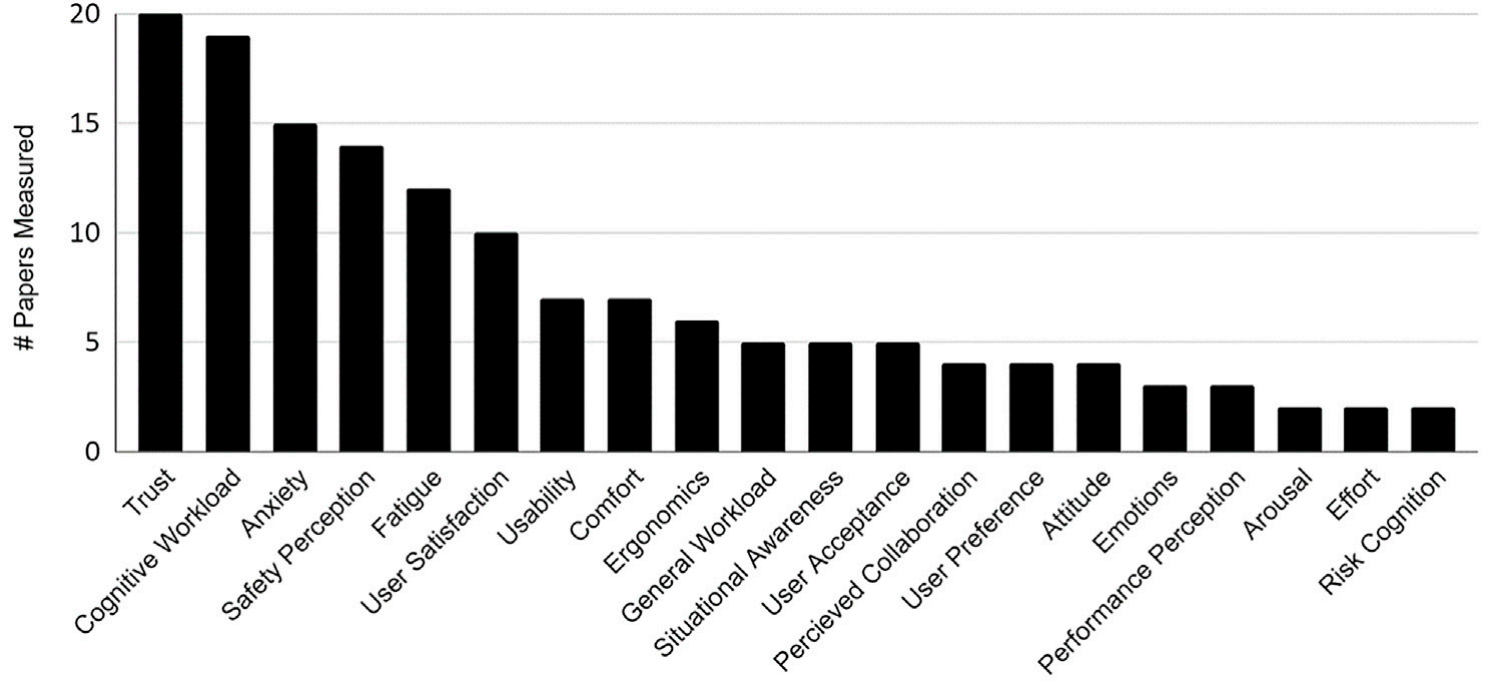
The popularity of human factors in shared-space human-robot collaboration measured out of 61 total papers.

A mean of 2.47 (1.22) human factors were examined per paper. Because some studies use different terms for a HF, but were referring to the same factor, each paper was thoroughly reviewed for how they introduced and defined the factor, the methods they used to measure the factor, and their terminology in order to use one universal term across multiple studies. Groupings were only formed for identically defined HFs with equivalent measurement goals. For example, the “anxiety” group included psychological stress and anxiety rather than solely using papers that explicitly mentioned the term “anxiety” only because these papers discussed this factor similarly and used the exact same surveys to quantify stress as they did anxiety. Trust in collaborative robotics was the most frequently measured human factor (*n* = 20 of 61), followed by cognitive workload (*n* = 19 of 61), and anxiety (*n* = 15 of 61) (Figure 2).

#### 3.3.1 Pertinent Human Factor Descriptions for Shared-Space Human-Robot Collaboration

##### 3.3.1.1 Trust, Safety Perceptions, and Robot Reliability Perceptions and Their Impact on Operator Acceptance and Reliance

The reliability of the cobot can influence the utilization and corresponding performance behaviors of the operator, and reliability is a consistently validated factor that influences operator trust (Chen et al., 2018), (Wu et al., 2017). Furthermore, the performance of the operator, when placed in a supervisory position, has been shown to be inversely related to the cobot’s reliability (Oakley et al., 2003). As the perception of reliability increases, humans tend to be more trusting of the cobot thereby increasing their reliance on the technology and willingness to allocate tasks (Charalambous et al., 2016). The relevance of safety perceptions, beyond robot reliability alone, has an evident impact on trust (Maurtua et al., 2017), (Lasota and Shah, 2015; Palmarini et al., 2018; Hald et al., 2019). As there are currently no universal definitions of trust, it has been priorly shown that trust definitions and perceptions differ between automation technology types, where trust in shared space robotics has a greater relevance of safety than trust in “automation” (Hopko and Mehta, 2021).

With increased levels of trust, operators tend to increase their reliance on cobots, choose to allocate more tasks to the cobots, and reduce supervision of cobots (Charalambous et al., 2016), (Lee and See, 2004), (Adnan et al., 2018), (Naneva et al., 2020). This allocation of tasks onto the cobot may reduce operator situation awareness by reducing engagement and distancing the operator from the loop. In such cases, over trust in the cobot can result in the misuse and abuse of the cobot with the operator neglecting attention to the robot and task (i.e., becoming distracted during the task or reducing maintenance on the robot outside the task), misusing the cobot outside its intended use, or overutilizing the cobot beyond its capabilities (Lee and See, 2004), (Hancock et al., 2011). In contrast to overtrust, undertrust can cause operators to reject adoption of cobots, and can cause significant discomfort to the operator resulting in decreased use or complete rejection of the cobot regardless of its capabilities. As such, the initial adoption of cobots has been shown to face barriers due to low acceptance by some assembly workers of cobot technology (Fletcher et al., 2017), (Meissner et al., 2020).

Trust and acceptance in technology-agent teammates have different fundamental constructs than with human teammates (Madhavan and Wiegmann, 2007). Interpersonal trust is founded on three bases, the ability—quality of skill, integrity—honest or lawful characteristic, and benevolence—the kindheartedness or Goodness of the teammate (Colquitt and Salam, 2015). Cobots differ from human teammates as they lack intentionality. They are not capable of developing their own intents behind their actions (Charalambous et al., 2016), (Madhavan and Wiegmann, 2007). Thus, cobots cannot be *good beings* and do not intentionally deceive people. While the ability of the cobot has been argued to be the primary basis of trust in cobots (Chen et al., 2018), operators are able anthropomorphize technology and instill a sense of malevolence (Nass and Moon, 2000). This is especially relevant in cobots that directly parallel human-like characteristics or in cobot systems susceptible to cyber intrusions.

The quality of human-robot collaboration is also related to the hierarchy between the operator and cobot, where trust based on the cohesion of teaming is increasingly relevant when the operator and cobot are at the same level in decision making verses the cobot being used as a tool (Chiou and Lee, 2021). With increasing level of collaboration between the human and robot, it is imperative to design robot behaviors and decision making with respect to how the user will perceive the safety and reliability of the system and their resulting trust. To satisfy human’s expectation of the behavior of the robot, provide a more comfortable and safe working environment, and calibrate operator’s appropriate utilization of the cobot, such attitudes towards cobots must be addressed.

##### 3.3.1.2 Cognitive Workload and Its Relation to Operator Fatigue and Anxiety

It is similarly necessary to consider the impact of a cobot teammate on the operator’s workload. The Yerkes-Dodson law illustrates that operator performance is directly related to the cognitive arousal of the operator, where hyperarousal, or overload, is associated with stress and anxiety, and under-arousal is associated with sleepiness and task disengagement behaviors. Designing tasks that result in operator overload can directly lead to decreased operator satisfaction and increased stress and anxiety levels (Rubio et al., 2004). Not only can hyperarousal be harmful to workers, it can also lead to increased operator error with damageable outcomes in operation. In contrast, underloading the operator can lead to boredom and task disengagement, which can similarly reduce task performance by increasing changes of slips or lapses (Rubio et al., 2004).

It is important to neither overload nor underload the operator in order to maximize operator performance and engagement. Collaboration requires the operator and cobot to jointly understand the capabilities of the other and to make decisions to allocate or share tasks accordingly. A more organic and effective team structure, i.e., appropriate workload allocations to humans and robots in a collaborative task, will facilitate fluent human-robot interactions (Chiou and Lee, 2021). Previous work has shown that inconsideration to these effective allocations can result in increased operator workload with corresponding performance decrements (Hopko et al., 2021a). Designing for appropriate workload is especially important because workload not only impacts instantaneous performance, but sustaining of overload can result in mental or physical fatiguing that can also reduce efficiency, decision making capability, and performance of the system as time-on-task increases (Peternel et al., 2018a; Li et al., 2019; Hopko et al., 2021a). Proper management of workload and appropriate allocations can improve performance and safety (Baxter et al., 2018). As humans and robots become more collaborative, the fluency of these interactions will become more pertinent considerations. The use of collaborative robots can often be designed to offload work from the operator onto the robot; however, the introduction of cobots itself can increase the cognitive loading on the user as more complex tasks may be introduced or require more situation awareness cognitive resources to perform the task (Hopko et al., 2021a). Such loading must be considered in order to prevent fatiguing of the operator, anxiety, and performance reductions.

##### 3.3.1.3 Situation Awareness, Vigilance, and Arousal and Their Interrelation With Trust, Workload, and Fatigue

An essential human factor consideration in collaborative robotics is the operator’s situation awareness (SA), which has similarly been shown to impact system safety and performance (Naderpour et al., 2015), (Kaber and Endsley, 2004). SA is the resulting mental model or state of knowledge about the system, or the situation. As such, SA impacts information processing and decision making as a result of vigilance towards relevant factors within the system (Endsley, 1988), (Smith and Hancock, 1995). Three time-dependent components of SA include perception, comprehension, and projection (Endsley, 2015). Perception refers to the ability to gather information, such as identifying the resources available and the state of the cobot. Comprehension is the understanding of the gathered information including evaluating the ability of the cobot’s current state to meet its goal. Lastly, projection is the ability to predict the next state of the cobot or the impact of the current state on the HRC’s goal. Thus, SA directly impacts the types of behaviors and decisions that are made based on the available information, cognitive resources, and attention on task.

In sense of behavioral cognition, performance degradation in sustained monotonous tasks or those with rare error rates are often present due to vigilance decrements, highly interrelated with operator fatigue, workload, situation awareness, and trust (Hancock, 2017). The inability to provide attention to relevant cobot factors (thus lower SA) can make it more difficult for operators to regain control over the system (Endsley and Garland, 2000), (Gombolay et al., 2017) or identify alarms or potential mishaps (Dixon et al., 2007). Lower SA can directly impact the safety of shared-space HRC by pulling the operator out-of-the-loop, such as the operator not tracking the physical position of the cobot increasing the likely hood of collisions.

##### 3.3.1.4 Pertinent Demographic and Dispositional Human Factor Considerations: User Age and Sex

The average age of machinery manufacturing workers in the United States is 44.7 years with 8.8% between 16 and 24 years, and 20.9% between 25 and 35 years of age (CPS, 2020a). This age distribution has been reasonably consistent for the past 10 years with median age at 44.6 in 2011, 44.9 in 2013, 45.8 in 2015, 45.3 in 2017 (CPS, 2020b), suggesting the projected age distribution to stay similar in the near future. Previous studies have validated that age is a driving factor in trust in automation and robots; older individuals tend to be less trusting than younger individuals (Schaefer et al., 2014), (Scopelliti et al., 2005). Moreover, age impacts cognitive workload perceptions and capabilities (Lee et al., 2009). As cobot operators encompasses a range of ages, the effects of age must be considered. Furthermore, the sex distribution of machinery manufacturing workers has steadily had a 30% female workforce (U. C. Bureau, 2020). The perception of cobot capabilities, effect of behaviors, and proxemic spacing has been shown to impact males and females differently, and in some scenarios, has been shown to be a larger factor than age (Syrdal et al., 2007; Kuo et al., 2009; Nomura, 2017). Due to social behaviors and social norms of males and females, the perceptions, values, and acceptance of cobots vary (Mutlu et al., 2006), (Hopko et al., 2021b). Additionally, workers’ pre-experience is found to be a crucial factor that influences workers’ states (Hopko et al., 2021b), (Wurhofer et al., 2015). Increased experience and familiarity with cobots results in higher levels of acceptance and trust, but can be biased by system reputations, recent publicized accidents, and preexisting expectations (Hoff and Bashir, 2015). Age and experience interplay in multiple dynamics: older generations have less experience with newly developed technologies (Kuo et al., 2009), thus are less likely to be familiar with cobots than younger generations. However, older generations have more general experience and cognitive biases than younger generations.

### 3.4 Human Factor Measurement Methods

The methods to quantify to the top five most frequently considered HFs, trust, cognitive workload, anxiety, safety perception and fatigue are summarized in Table 3. Methods were split by relationship type, with either a primary or secondary relationship. Primary types are direct measures of the state and secondary types are peripheral measurements that were claimed to have strong relationship to the operator state, even if not specifically measuring it. Methods were also split by subjective or objective methods. Subjective methods capture the operator’s perceptions and first-hand experience. Objective methods are obtained through sensors, observation, or modeling.

**TABLE 3:** Human factor measurement methods.

	Subjective Measures		Objective Measures	
	Primary	Secondary	Primary	Secondary
Trust	13 Trust Survey 3 Free Response & Interviews	4 Performance Survey2 User Satisfaction Survey 2 Safety Perception Survey1 Neg. Attitude Towards Robots1 Risk Taking Attitude1 Robot Predictability Survey	3 Modeling1 Intervention Rate 1 Operator Body Pose	3 Robot and Human Performance 2 Human Fatigue Level
Cognitive Workload	14 Cog. Workload Survey 1 Think Aloud Protocol 1 Interviews	3 User Satisfaction Survey 1 Safety Perception Survey 1 Difficulty Perception Survey 1 Ease of Monitoring Survey	2 Eye Tracking 2 Modeling 1 Electroencephalogram (EEG) 1 Electrocardiogram (ECG)	1 Robot and Human Performance
Anxiety	8 Anxiety Survey 1 Self-Reporting	3 Discomfort Survey 2 Neg. Attitude Towards Robots 2 User Experience Survey 1 Risk Perception Survey 1 Safety Survey	6 Electrodermal Activity (EDA) 3 Electrocardiogram (ECG)	1 Hand-Eye Coordination 1 Eye Tracking
Safety Perception	11 Safety Survey 2 Interviews 1 Think Aloud Protocol	3 Robot Capability Survey 3 Trust Survey 2 User Acceptance Survey 2 Perceived Ergonomics	1 Human Separation Distance 1 Electrocardiogram (ECG) 1 Electrodermal Activity (EDA)	1 Human Intervention Rate
Fatigue	1 Tiredness Survey	1 Ease of Monitoring	8 Force Myograph (FMG) 5 Electromyogram Activity (EMG) 3 Modeling 2 Endurance 1 Speed of Human Adaptation 1 Critical Flicker Frequency	1 NIOSH Standards

The top five measured HFs, are represented above with corresponding # column representing how many papers use this method.

#### 3.4.1 Trust Measurement Methods

The vast majority of the trust literature used Likert-scale questionnaires to quantify trust (*n* = 13 of 20), including the trust in automation questionnaire by Jian et al. (Jian et al., 2000), trust in robots by Dragan et al. (Dragan et al., 2015), and by Körber et al. (Körber et al., 2015). The remainder of the studies either developed their own questions or did not provide any information on the questionnaire they used. In addition to trust questionnaires, six other questionnaires were used to capture secondary measures of trust: safety perception, robot predictability, performance perception, negative attitude towards robots, risk-taking attitude, and user satisfaction.

For objective measures, three of the papers used mathematical models to directly quantify trust (Chen et al., 2018), (Wu et al., 2017), (Rahman and Wang, 2018). All of these papers used models with trust as the response that relied on real-time human and robot performance as the input. (Rahman and Wang, 2018) developed a time series linear model using both human and robot successes and mistakes in a Lego assembly task, where the human and robot alternate to place pieces on the structure. A successful action of the robot improves the response (human trust) by one unit, and a mistake reduces trust by one unit. Another included study developed a Markov decision process with a probability of moving to a higher/lower trust and fatigue state dependent on robot performance and trust repair time measured during a collaborative assembly process (Wu et al., 2017). The study reported that a first-order approximation using robot performance can accurately model human trust, where trust moves one state higher/lower at a time. This assumption was also mentioned by Chen et al. (Chen et al., 2018) who also used a Markov model with a state space that represented previously identified human trust/belief states. Movement to a higher/lower trust state is dependent on the history of the robot’s performance of all previous interactions rather than only the last interaction. In addition to a partially observable Markov decision process, Chen et al. also recorded the intervention rate of the operator taking over a subtask, with more interventions for risky subtasks. Operator pose and gestures have also been used as a measure of trust (Hald et al., 2019)—where reactive movement were distinct between trustworthy and untrustworthy conditions.

#### 3.4.2 Cognitive Workload Measurement Methods

Similar to trust, cognitive workload was primarily measured using subjective questionnaires (*n* = 14 of 19). The most popular questionnaire was NASA Task Load Index (TLX) utilizing both pairwise comparison or raw TLX rankings (Hart and Staveland, 1988) (*n* = 12 of 14). Another widely-recognized questionnaire is a one-dimensional Rating Scale of Mental Effort (RSME), which is stated as a one-dimensional version of TLX that only measures mental effort (Ghanbary Sartang et al., 2016). Additional primary subjective methods included the think-aloud protocol and interviews (*n* = 1 of 19 for each) (Materna et al., 2018). Secondary questionnaires were also employed to capture similar or subset states including user satisfaction, safety perception, difficulty perception, and ease of use, all discussed as impacting workload or workload perceptions.

Objective methods to capture cognitive workload included bioinstrumentation, performance decay, and mathematical representation. Bioinstrumentation included EEG brain monitoring—where increased cerebral cortical activation in the brain correlated with higher levels of cognitive workload (Amirhossein and Ehsan, 2019), eye tracking—where average fixation time and pupil dilation were evaluated (Tang et al., 2019), (Kuz et al., 2018), and ECG heart rate monitoring—where respiratory sinus arrhythmia (RSA) was calculated, but heart rate features were not explicitly reported (Kato et al., 2010). Memar and Esfahani (Amirhossein and Ehsan, 2019) jointly utilized subjective and objective measures evaluating TLX and EEG. By using the extracted spectral power density and coherence features from EEG, they developed a cognitive workload classifier. Additionally, they found the result of TLX is consistency with the result of the EEG-based workload observation.

Two included articles used mathematical representation papers: one used a Markov decision process to simulate how human cognitive state transitions from the current state to next state with the consideration of observation, estimated by human “belief” parameters (Buehler and Weisswange, 2018). The second paper used a simulation model (Rabby et al., 2019), which simulated: 1) human physical performance based on two parameters, namely stand for muscular contraction, and expansion for system and muscles’ fatigue level and their recovery; 2) a human cognitive workload model based on parameters that represent the complexity of tasks and the participants’ utilization factor; and 3) a human cognitive performance model based on estimated parameters of cognitive workload, physical workload, maximum cognitive performance, and additional workload due to robot’s mistakes.

#### 3.4.3 Anxiety Measurement Methods

The primary method to capture anxiety was subjective questionnaires including the state trait anxiety inventory questionnaire (STAI; *n* = 2 of 9) (Spielberger, 2010), semantic differential (fear, surprise) questionnaires (*n* = 5 of 9), and robot anxiety survey (RAS; *n* = 1 of 9) (Nomura et al., 2006), or self-developed questionnaires (*n* = 1 of 9). Additional related questionnaires included negative attitude towards robots, user experience, risk perception, safety perception, and discomfort.

The ECG features found to correlate with anxiety and stress included an increase in standard deviation of the sympathetic band (Sims et al., 2002), and decrease in the frequency of the parasympathetic band (Sims et al., 2002). Another included study did not find a difference in ECG response between the stress and no stress condition (Eimontaite et al., 2019). Variation in skin conductance level was also evaluated but no significant findings are reported (Etzi et al., 2019). Skin potential reflex features included the amplitude and frequency of response—where higher amplitude and frequency correlated with higher stress (Fujita et al., 2010), (Kato et al., 2010), (Arai et al., 2010), (Tan et al., 2010). Eye tracking features included gaze duration—where longer duration was associated with improved graphical signage potentially reducing mental strain (Eimontaite et al., 2019).

#### 3.4.4 Safety Perception Measurement Methods

The majority of safety perception questionnaires were encapsulated with trust questionnaires (Palmarini et al., 2018); (Baxter et al., 2018). Additional questionnaires were either unspecified or developed by the researchers (Liu et al., 2016). Safety perception has also been recorded using think-aloud protocols and interviews (Materna et al., 2018). Peripheral surveys included: trust, robot capability, user experience and perceived ergonomics of the task.

Furthermore, many studies measured anxiety and safety perception jointly; therefore, the ECG and EDA data follow the same trends as anxiety measures—where increased sympathetic activity corresponds to lower perceptions of safety or increased amplitude and frequency of skin response corresponds to lower perceptions of safety (Weistroffer et al., 2013). Other safety perceptions were measured by the human’s behavior and included the number of human interventions during “risky” decisions by the robot or the average distance the human maintains between the cobot and themselves (Lasota and Shah, 2015).

#### 3.4.5 Fatigue Measurement Methods

In contrast to other HFs, fatigue assessment was primarily measured objectively. The only paper that used subjective measurements of fatigue employed a tiredness questionnaire with 7-point Likert scale (Tang et al., 2019). The majority of the included papers (*n* = 11 of 12) measured neuromuscular fatigue or fatigue owing to unspecified causes, rather than cognitive fatigue. For those that measured neuromuscular fatigue, many utilized muscle fatigue data through change in EMG signal frequency domain or utilized FMG: increased estimated mean EMG amplitude and decreased mean frequency correlates with muscle fatigue (De Luca, 1984; Peternel et al., 2018b, 2019; Lorenzini et al., 2019). (Rahman and Wang, 2018) proposed a human performance model with the consideration of muscle fatigue and recovery—where fatigue was quantified using a human’s speed parameter. Fatigue was also modeled using a Marcov decision process with a probability of moving to a higher/lower fatigue level based on time-on-task and repair time (Wu et al., 2017), or through direct modeling—where fatigue exponentially increases with working time (Li et al., 2019). NIOSH standards or joint torque calculations to identify fatigue progression have also been applied (Lorenzini et al., 2019), (Pini et al., 2016).

### 3.5 Identified Effects of Human Factors on Metrics of Human Factor Consideration

#### 3.5.1 Quality of Task and Related Performance Metrics

Based on the systematic review, it was observed that many HFs impact the collaborative performance of the HRC system including cognitive workload, fatigue, trust, anxiety, and safety perception. This subsection reports the impact of HFs on aspects of system performance, accuracy, and quality, as observed in the systematic review. The included studies identified an effect of cobot behavior on anxiety and safety perception, with resulting system-wide performance decreasing as anxiety increases (Koppenborg et al., 2017), (Oyekan et al., 2019) or as algorithm transparency decreases (Baxter et al., 2018). Anxiety also induced attentional decrements (Oyekan et al., 2019). System performance was found to correlate with operator cognitive workload: reduced cognitive workload by use of graphical signage resulted in improved system performance compared to control (Eimontaite et al., 2019). When simulating a cognitive performance model, cognitive workload was used as the primary factor, which implicated the impact of cognitive workload on the performance (Rabby et al., 2019). Over time, operator fatigue levels were found to increase with respect to time-on-task, and increased levels of fatigue resulted in reduced system performance (Wu et al., 2017), (Li et al., 2019), (Peternel et al., 2018a), (Anvaripour et al., 2019). Cobot adaptation to physical fatigue was shown to reduce the effort the operator requires to maintain collaborative performance (Peternel et al., 2018a), (Peternel et al., 2019). However, operator task preferences were shown to directly impact performance, such as task completion speed, short term accuracy, and long term accuracy (Zhao and Pan, 2018), with adaptive robotics.

Trust in the cobot was also shown to impact system performance—where larger perceived trust results in higher productivity, team fluency, and manipulation quality during the HRC (Rahman and Wang, 2018). With higher (preferably optimal) values of trust, the teaming aspect of HRC improved, with the collaborative robot being perceived more as a team member than as a tool (Rahman and Wang, 2018). Beyond optimal levels of trust, vigilance and focus of the operator declined that in turn induced negative performance and impacted system accuracy, as measured by a decrease in gaze duration on the task (Eimontaite et al., 2019). Furthermore, trust was found to be influenced by the operator’s experience and level of training only when the trainee was invested (Robert and You, 2016). Training also improved performance when operators had lower control.

#### 3.5.2 System Efficiency and Fluency

Operator manipulation speed has been shown to decrease as anxiety and mental strain increase (Tan et al., 2010), owing to increased cobot movement speeds (Charalambous et al., 2016), (Etzi et al., 2019), (Koppenborg et al., 2017), (Henriksen et al., 2020). The impact of cobot speed was also found to influence the perception of team-fluency, with improved team-fluency when speeds are slower (Koppenborg et al., 2017). This effect can partially be attributed to the lack of transparency behind the intents of the cobot, or perceptions of poor safety caused by the movements, where two studies have found that operators have a tendency towards longer response times when the transparency of the system is low (Etzi et al., 2019), (Koppenborg et al., 2017). Operators were found to be more comfortable with ‘human aware’ cobots, i.e., a cobot that actively tries to predict the next action of the human (Lasota and Shah, 2015). Additionally, system performance and efficiency significantly improved due to operators reducing lead times, changing their behavior to increase concurrent movement with the cobot, and reducing task execution time (Lasota and Shah, 2015). However, the resulting impact of this adaption was shown to cause operators to have higher cognitive load in an attempt to achieve system efficiency (Lambrecht and Nimpsch, 2019).

The employment of manual, reactive or predictive strategies to interact with a collaborative robot was found to result in variance in cognitive workload, temporal stress, and resulting efficiencies and fluency (Lambrecht and Nimpsch, 2019), (Sadrfaridpour and Wang, 2018). A tradeoff of minimizing down time in a system and minimizing strain has been observed (Pearce et al., 2018). Similarly, task efficiency was improved when the weight of objects picked up by the operator were lighter, and efficiency was dependent on the speed of the cobot which has an optimum trade-off with efficiency (Rahman and Ikeura, 2018).

#### 3.5.3 Acceptance and Operator Utilization Strategy

User satisfaction and acceptance of the technology impacts how operators choose to utilize the technology in both the initial adoption and continual use with the device. Increased trust in the system was shown to result in a higher rate of utilization (Rahman and Wang, 2018). Moreover, operators were less likely to intervene in the cobots task when trust and familiarity with the system were high (Chen et al., 2020). Safety, robot performance, and amount of information provided significantly influenced trust perceptions (Palmarini et al., 2018) and willingness to adopt cobot technology (Maurtua et al., 2017). In some studies, trust was inversely correlated with cognitive workload (Rahman and Wang, 2018), (Sadrfaridpour and Wang, 2018); the increased trust through understanding of the collaborative system led to reduced cognitive workload and higher situation awareness (Rahman and Wang, 2018). Participants, in general, were found to prefer more transparent systems (Rahman and Wang, 2018).

In a survey of manufacturing workers, safety perception was identified as the most important theme in predicting trust in a cobot, as the workers’ main fear was getting hit by the robot (Charalambous et al., 2016), (Maurtua et al., 2017). Many workers stated that their trust directly impacted their mental models when interacting with a cobot. During a think-a-loud protocol involving the study of trust in cobots, participants’ most discussed theme was the performance of the cobot, while physical robot attributes did not receive much attention (Nahmad Vazquez and Jabi, 2019).

## 4 Discussion

In this paper, we conducted a systematic review to evaluate the impact of most commonly examined human factors (HFs) in shared-space human-robot collaboration (HRC), document methods to analyze these HFs, and discuss how these factors impact aspects of HRC such as system performance, efficiency, teaming, and utilization. The key findings are:

1) The most studied human factors include trust, cognitive workload, and anxiety, with the most popular HF assessment methods being subjective questionnaires.
2) Human factors directly impact system performance, efficiency, acceptance, and other components of HRC; however, most studies limit their discussion to the impact of the robotic system on human factors, but few emphasize the resulting impact of human factors on the system or manipulate human factors directly. There are even fewer studies that consider both relationships (i.e., human-to-robot factors, and vice versa) for considerations of closed-loop HRC designs.
3) Most studies used skewed sample demographics or fail to report relevant demographics, where studies utilize more male than female participants, more younger than older populations, and often fail to report participant’s prior experience and experimental training methodology—which may result in a partial understanding for workforce development strategies with collaborative robotics.

### 4.1 Model of Collaboration-Centered Design

**FIGURE 3:**
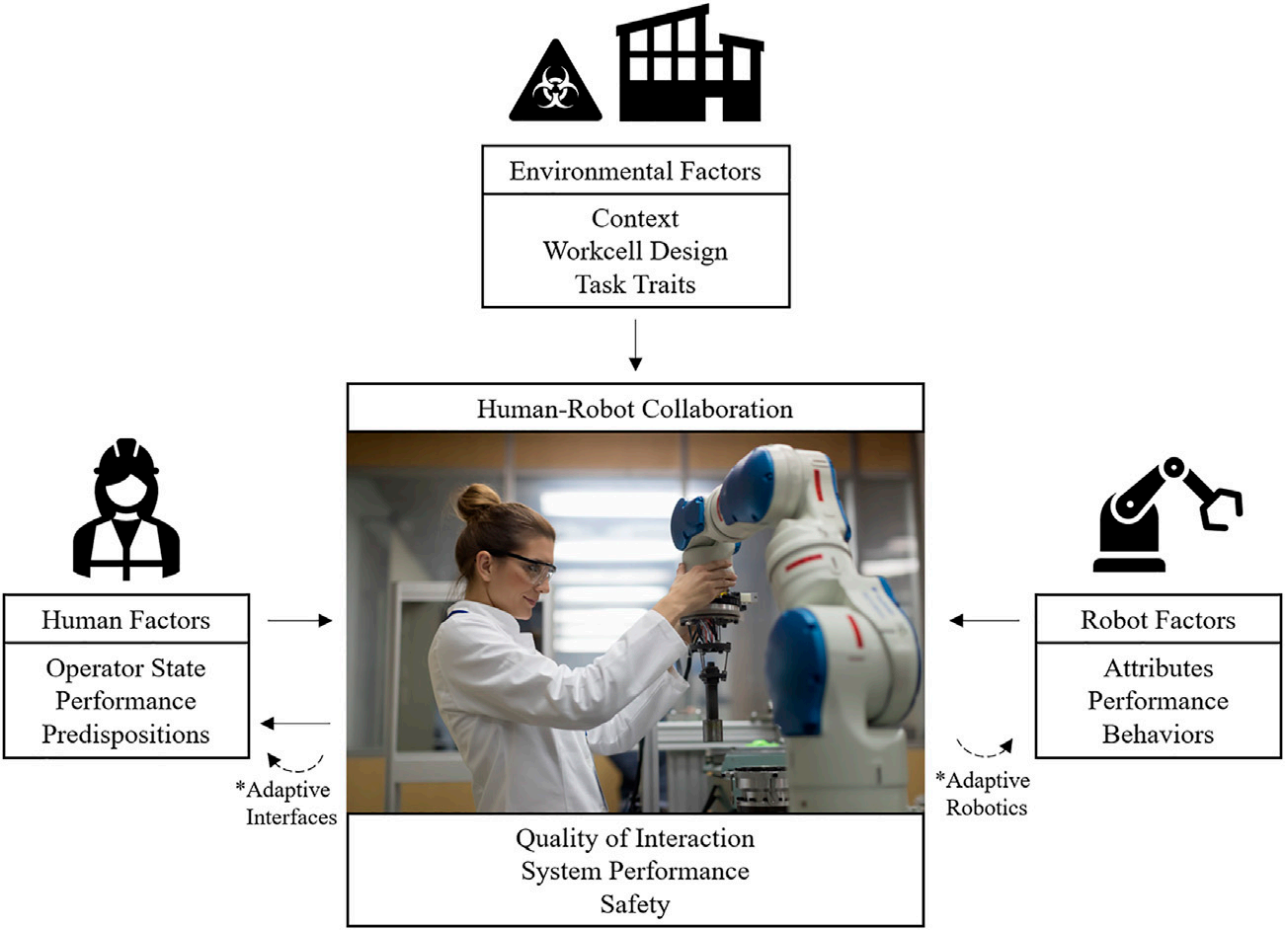
Directional effect of factors in HRC as identified in literature review. Note. Listed subfactors are examples rather than an exhaustive list.

A collaboration-centered concept map is illustrated in Figure 3 based on identified influences found in the literature search. While the scope of HRC varies between studies, we define HRC to include the entire human-robot system and environmental, robot, and human context. Thus, the emerging factor that is HRC is influenced by environmental factors (EF; such as task traits, context, and workcell design), robot factors (RF; such as reliability and automation), and human factors (HF; such as operator states, attributes, and experience). Traditional manufacturing robotics influence HRC without feedback; meaning traditional robotics require the operator to adapt to aspects of HRC in place of the robot (Chen et al., 2018). With the use of more collaborative robotics additional sensors allow for information about the state of the HRC system including online context of HFs (Lee and Seppelt, 2009); (Chiou and Lee, 2021). Unlike cobots, which require intentional programming and sensors for specific feedback capabilities, HFs are naturally cyclic as they influence the quality of HRC and adapt to HRC. This natural loop allows for the advantage of operator sensing capabilities in HRC, but it also implies that the design of HRC systems directly influences how operators perform in the system, the tendencies to trust the system, etc. The influence of HRC systems on human factors can also be modulated through the use of adaptive interfaces, such as augmented reality technologies, sensor feeds, or others. Unlike human or robot factors, no included studies adapted environmental factors to the state of the HRC system, likely because in shared-space robotics, the tasks tend to be monotonous, and environmental factors, such as emergency lights tend to be attributable to the cobot. Future work may consider a potential HRC adaptation based on environmental factors rather than robot factors alone.

The joint consideration of EFs, RFs, and HFs as they interact in HRC result in the success or failure of the HRC system emergent factors (i.e., performance level, safety, acceptance). Due to the existence of the HFs-HRC loop, it is important to consider both the implication of human factors on the HRC system and the implication of the HRC system on human factors. RFs can influence HFs indirectly, through manipulating an aspect of HRC. The relationship between human, robot, and environmental factors has been previously modeled, most often in the trust literature (Hancock et al., 2011). Understanding the feedback aspect of the HFs-HRC loop and mechanism of influence of HFs in the proposed model is often overlooked in research studies.

### 4.2 The Need for Robust Metrics to Capture HFs and Their Interactions

The use of subjective and objective measurements is important to accurately quantifying the effect of HFs on HRC. Subjective measurements, which were observed to be more commonly employed, provide implicit information as to how, why, or what an operator is experiencing; however, due to the discrete nature of most subjective measurements, the dynamic interpretability of the states is harder to capture (Jahedi and Méndez, 2014). In contrast, objective methods are often able to provide explicit continuous measurement, thus provide additional insight into the mechanistic influences of the HFs on HRC, right when they occur. Serve as a whole, the perceived responses and physiological and behavioral responses collectively offer a comprehensive perspective of human factors. In this way, the use of both can make a study of human-robot collaboration more integral. We observed that objective methods, through the use of bioinstrumentation, human behavior observation, or mathematical modeling are infrequently applied to trust, workload, and safety perceptions. In contrast, fatigue measurement and anxiety perception are more commonly measured objectively, allowing for direct modeling of the interaction between HFs, RFs, and EFs.

Trust calibration, reduced anxiety, optimal cognitive workload, fatigue mitigation, and improved user satisfaction are all desirable in ideal cobot design. There is potentiality for many of these states to be covariates manipulated by EFs and RFs. The influence of EFs such as the positioning between the operator and cobot or type of task being performed can influence how the operator reacts to RF manipulation (Hald et al., 2019), (Rabby et al., 2019). Furthermore, the majority of the included papers measure trust, cognitive workload, and anxiety, which have promise to be interrelated components in HRC. Frequently measured states alongside trust include participants’ attitudes and safety perceptions, and safety has been shown to be a significant influence on trust in cobots (Charalambous et al., 2016); (Palmarini et al., 2018). In fact, many questionnaires, both validated or developed by researchers to quantify trust, use questions related to the predictability or safety perception when using the cobot. Thus, where possible, future work is warranted that delineates these HFs, controlling for potential covarying responses or reporting relationships between states.

There is also a need for robust dispositional and demographic reporting and training methodologies. Over 78% of the included papers failed to report the experience level of their participants with cobots and over 70% failed to report how they were training their participants for the experiment. Operator experience has historically been shown to be one of the larger predictors of how the operators will trust and utilize the technology (Hopko et al., 2021b), thus such a large portion of the literature not reporting their training methodology or key demographic information leads to partial understanding of human factor considerations. Beyond experience, 52% of the studies failed to report age of their participants, 43% to report gender distributions, and 20% failed to even report the sample size of their population. Future human factor research requires more rigorous sampling and methods reporting in order to fill the gaps on how these dispositional human factors impact other human states, such as trust and workload perceptions, as well as metrics of human-robot collaboration, such as performance and utilization.

### 4.3 Considerations for Improved Human-Robot Collaborations

The review emphasized the influence of robot factors and attributes, including the design of robotic manipulations, interaction processes and subsequent performance, as well as algorithm behaviors, on human states and overall system success. However, we identified a critical gap in the examination of important human factors owing to limitations in study design, participant inclusion, and experimental methods. To fundamentally understand the design of collaboration centered HRC systems, the effects of HFs and RFs *in context* cannot be ignored. This section discusses the effects of robot design, participant demographics, measurement methods, and HFs jointly to provide recommendations for improved HRC.

#### 4.3.1 The Impact of Robot Behavior Dynamics

Robot behavior has been shown to manipulate HFs where increased movement speed (Hald et al., 2019), more dominant movements (Reinhardt et al., 2017), lack of predictability, or inadaptability of the robot (Nikolaidis et al., 2017a), (Nikolaidis et al., 2017b) can decrease trust perceptions and/or increase anxiety. Maximizing trust or minimizing anxiety are not necessarily ideal goals, as each extreme (i.e., very high or very low) can result in undesired or unintended human behavior (Lee and See, 2004). Within the HRC domain, trust and anxiety are often shown to have inverse correlations (Gillath et al., 2021), (Miller et al., 2021). Similarly, increased trust is accompanied by decreased cognitive workload (Sadrfaridpour and Wang, 2018), (Mizanoor Rahman and Ikeura, 2018) and decreased frustration (Hamacher et al., 2016)—a component of workload perception (Hart and Staveland, 1988). Future work needs to delineate relative contributions of each of these interrelated states (trust, cognitive workload, anxiety).

#### 4.3.2 The Impact of Robot Ability and Performance

Improving robot ability will directly improve system performance during nominal operating conditions. Higher reliability levels can reduce operator cognitive workload (Rabby et al., 2019). Reduced operator cognitive load can further improve system performance as the onset of fatigue is slower and fatigue recovery can implement with automated processes (Hopko et al., 2021a). Highly reliable systems and/or monotonous collaborative systems can, however, reduce operator engagement in the task effectively decreasing situation awareness or causing complacency, often synonymous with overtrust (Hancock, 2017). This disengagement from the task in highly reliable situations may partly attributed to lower operator cognitive arousal (Kompatsiari et al., 2019), which may result in vigilance decrements or resource re-allocation (Smith and Hancock, 1995), in addition to reduced effort to maintain performance by the operator. Directly maximizing robot performance will not necessarily maximize HRC’s system output performance—a premise requiring acknowledgement and acceptance in robotics design. Robot performance is one of the leading factors that manipulates human trust in a cobot, where high robot performance directly corresponds with high levels of trust (Rahman and Wang, 2018), (Hald et al., 2019), (Nahmad Vazquez and Jabi, 2019), (Nikolaidis et al., 2015). When trust is too high, operators can become complacent, continue using the cobot after signs of unreliability, stop monitoring the cobot, or other undesirable behaviors. Providing cognitive support for such cases, potentially through the use of augmented or mixed reality environments or training may address performance concerns.

### 4.4 Opportunities for Future Work

The impact of dispositional factors on successful HRC is complex, and critical to document, but significantly understudied. This is likely a major barrier to safer and more use-inspired robotic assistance in shared space HRC. Factors such as age, gender, culture, and personality, can impact how operators perceive cobot behavior (Hancock et al., 2011), but were understudied. For example, attitudes and emotions were examined by less than five studies, and none of those included papers discussed gender or age effects in HRC. Not only were these factors not examined formally, the lack of consideration of these human factors were even more evident in those studies that failed to document the age or gender of their participants (which was 52.45 and 42.6% of the included papers, respectively). The introduction of cobot technology can disrupt traditional workforce norms and procedures, and the implication of the workforce’s age and gender can directly influence adoption in addition to the success of HRC long-term. More importantly, owing to their projected profound impact on workforce disruption and potential development strategies (Haden and John, 2021), it is imperative to understand the impact of key dispositional factors on aspects of HRC.

Recognizing that novice and expert operator statuses impact system performance and cobot utilization (Blanchet et al., 2019), the effects of prior operator experience, task familiarization, and training methods on HRC are understudied. For example, 70.49% of included papers in the review failed to mention the training methods. For those that did report training, they mentioned (but not examined) the duration of HRC training, and a variety of methods and durations were used across studies. Experience impacts perceptions of task difficulty, cognitive workload, trust in the cobot and other human factor, thus it is imperative that studies, at the very minimum report participant experiences and expertise levels, to offer transparency in how study findings could be applied in informing or evaluating robotic controls, interaction designs, or operator training strategies for HRC, etc.

Robotic utilization strategy and resulting system performance effects are understudied. The review highlighted a major focus on studies that examine the influence of robot factors on human factors (e.g., robot reliability impacts operator trust), but identified a major gap in research investigating the impact of human factors on overall system outputs (e.g., how trust impacts system performance or robot utilization strategy). Future research is warranted to systematically determine the singular and collective impacts of various human factors, such as trust, anxiety, safety perception, on system performance and technology acceptance/usage, and to determine the drivers of operator behavior changes with the robotic counterparts.

### 4.5 Study Limitations

The current systematic review focused on HRC factors for shared-space collaborative robot applications. Other forms of collaborative robots, such as mobile robots, were not included to capture the shared-space implications in HRC. As this paper focuses on HRC rather than robotics as a whole, this required search terms to be related to “human-robot collaboration”, thus papers that used other terms were not pulled. This limitation was partially mitigated by applying similar terms and related subjects within the EBSCO search feature. Heightened levels of vulnerability are present in shared space industrial-grade robots, and different types of physical human-robot interactions are required (Heinzmann and Zelinsky, 2003). It was important to understand cobots distinct from other types of robots due to these new dynamics. Furthermore, purely social robotics were excluded from this review. Social robotics intentionally anthropomorphize robot attributes and behaviors, which can manipulate HFs differently and place different importance on RFs that non-social systems and tend to be used for different goals, thus were not included. The consideration of RFs, such as the size, visual design, and anthropomorphism may be relevant considerations for HRC tasks. As it is possible to have social robots in HRC, other studies should consider the impact of anthropomorphism in HRC given these findings.

## 5 Conclusion

This work systematically reviewed human factor (HF) literature in shared-space human-robot collaboration (HRC), the metrics to measure HFs, and the implications of HFs on HRC. We identified the most frequently studied states to include trust, cognitive workload, and anxiety, where subjective questionnaires are the most popular methods; however, the use of bioinstrumentation, objective behavioral analyses, and mathematical representation, have also been used in various papers. It was observed that the majority of studies discuss HFs as dependent variables manipulated by robot factors (RFs) or environmental factors (EFs). Limited work has been conducted on the reverse direction, i.e., the resulting impact of HFs directly on HRC metrics such as performance or fluency. Furthermore, not only is the impact of demographic factors (e.g., age, sex) understudied, more than half of studies do not even report demographic information of their participants. A similar shortcoming was observed with training methods, where less than 30% of studies report participant training, and less than 36% report the prior experience of their participants with collaborative robotics. These finding indicate that dispositional factors are woefully understudied and underreported. The systematic review was able to capture the essence of HF considerations and current metrics in shared space HRCs and the potential interaction between many HFs, thereby providing opportunities for system perspectives in HRC designs applications.

## Data Availability

The original contributions presented in the study are included in the article/Supplementary Material, further inquiries can be directed to the corresponding author.
